# Evaluation approach of prefabricated components based on multi-layer complex network model combined with improved topsis method

**DOI:** 10.1371/journal.pone.0322236

**Published:** 2025-05-07

**Authors:** Xizhen Xu, Qun Wang, Xiaoxin Ding, Tiebing Chen, Ronghui Deng

**Affiliations:** 1 School of construction engineering, Shenzhen Polytechnic University, Shenzhen, China; 2 School of Economics and Management, Beijing Jiaotong University, Beijing, China; 3 School of Economics and Management, Jilin Jianzhu University,Changchun,China.; China Construction Fourth Engineering Division Corp. Ltd, CHINA

## Abstract

Prefabricated buildings face greater and different prefabricated components throughout their entire lifecycle, leading to a significant increase in management difficulty. This article proposes an evaluation method based on a multi-layer complex network model combined with an improved topsis. Firstly, by combining relevant regulations, literature, and engineering experience, the factors affecting the connection of components in prefabricated building are identified and the relationships between these factors are clarified to construct a multi-layered complex network model. Secondly, complex network theory is applied to calculate and analyze the importance evaluation indicators of the model nodes. Finally, the nodes are evaluated using the entropy weight optimization topsis method, and key nodes are selected based on the comprehensive importance evaluation value, and simulation verification is carried out by attacking the nodes. A specific model is constructed and analyzed using a building in Shenzhen, Guangdong Province as an example. The study shows that:(1) By analyzing key importance evaluation metrics such as node degree, betweenness centrality, and closeness centrality, the critical nodes identified for the project are “G2,” “G1,” “S24,” and “Q1”; (2) According to the comprehensive evaluation results using the improved topsis method, quality issues are the core cause of connection problems in prefabricated components, with the construction phase being the peak period for such issues; (3) The critical nodes play a significant role in maintaining the coordination and robustness of multi-layer complex networks, and the failure of these critical nodes undermines the network’s cohesion and synergy. This study provides new insights and methods for the evaluation and management of prefabricated construction.

## 1. Introduction

### 1.1. Background

With the acceleration of urbanization and the increasing market demand, the scale of construction projects continues to expand. During the “13th Five-Year Plan” period, the added value of the China’s construction industry increased by an average of 5.1% annually, accounting for over 6.9% of the gross domestic product [1]. At the same time, the “14th Five-Year Plan for the Construction Industry” proposes to vigorously develop prefabricated building, aiming for prefabricated building to account for over 30% of new construction projects. The construction industry, as a pillar industry of the China’s national economy, continues to play an increasingly important role, making significant contributions to promoting economic growth in China [2]. Prefabricated building, assembled from prefabricated components, faces significantly increased construction management challenges as the prefabrication rate rises. This includes the secondary deepening collaborative design of a larger variety of prefabricated components, multi-party collaborative construction involving more stakeholders, and the massive collaborative information generated as a result [3, 4]. The increased construction management difficulty directly leads to a substantial rise in project costs, making it difficult to control project quality, duration, and even safety according to the planned schedule. This greatly constrains the development of prefabricated building [5]. For the tracking survey of prefabricated building construction process in recent years, it is found that the existence of some unfavorable factors restricts the promotion of prefabricated building, so the scientific and reasonable evaluation of the importance of the relevant indexes of the prefabricated building parts and components, timely detection and reasonable control of the key indexes are of great practical significance for the promotion of the sustainable development of prefabricated building [6].

### 1.2. Literature review

#### 1.2.1. Evaluation indicators.

Currently, risk assessment and quality evaluation in the construction of prefabricated buildings are hot topics in the field of construction evaluation research. Wang et al. evaluated the quality indicators of prefabricated buildings using a simulated annealing-optimized projection pursuit model [7]. Lu et al. established an evaluation model for construction risks in prefabricated buildings based on weights and catastrophic events [8]. Cao et al. assessed the risks of prefabricated construction under the general contracting model from the perspective of the general contractor using a structural equation model, and proposed countermeasures for key risks [9]. Other scholars have also evaluated different indicators. Chang et al. conducted a risk assessment of prefabricated construction based on Prospect Theory [10]. Wang et al. evaluated the comprehensive benefits of prefabricated buildings using NSGA-II and simulated annealing optimized projection pursuit [11]. Dogan et al. performed a comprehensive risk assessment of buildings in Manavgat, a region in the Mediterranean [12]. Fan et al. proposed a green assessment method for prefabricated buildings based on AHP-EWM [13]. Cheng et al. combined topsis with Prospect Theory and interval-valued Pythagorean fuzzy numbers to establish an evaluation model for prefabricated construction, providing a structured approach to enhance the efficiency and safety of prefabricated building projects [14]. From the above studies, it can be observed that the current evaluation research on buildings mainly focuses on individual evaluation indicators. As a result, the evaluation models established in this manner overlook the interrelationships among various evaluation indicators, which does not align with the intricate and complex practical situations in engineering [15, 16]. Furthermore, based on the different construction purposes and management modes of buildings, the importance of various evaluation indicators for buildings varies significantly at different stages of their lifecycle [17, 18]. As components of the building structure, the importance of evaluation indicators for prefabricated components also changes accordingly. However, the importance of the evaluation indicators for components also exhibits different states at various stages of their lifecycle [19].

#### 1.2.2. Evaluation method.

There is a significant amount of research focusing on scheme evaluation, with various methods being developed for this purpose [20]. Li et al comprehensively considered factors in building safety risk assessment and, using structural equation modeling and system dynamics modeling, established a model for safety risk assessment in prefabricated building [21]. Li et al. used structural equation modeling to study the three main risk types to assess the construction risk of assembled buildings and validated the SEM model using cloud modeling assessment [22]. Cai et al. developed a combined AHP-DEMATELISM model for assessing the risks in the prefabricated construction supply chain, utilizing the ISM model to hierarchically display the structural relationships of influencing factors [23]. Wang et al. established an evaluation model using gray relational analysis to assess and analyze textual data on incentive policies for prefabricated construction across various provinces [24]. Yang proposed a fuzzy neural network risk assessment method tailored for prefabricated construction, creating a robust and adaptive evaluation model for coarse prefabricated construction using fuzzy neural networks [25]. Wang et al. developed a risk evaluation index system for prefabricated construction and conducted sensitivity analysis of the probability of risk occurrence using sensitivity functions, effectively identifying risk factors [26]. With the continuous advancement of mathematical evaluation models, approaches such as the entropy weight-uncertain measure theory [27], entropy weight-rough set model, grey clustering [28], and entropy weight-fuzzy comprehensive theory [29] have gradually been applied to the evaluation research of prefabricated building, to a certain extent, promoting the development of prefabricated building evaluation research.

From the current research status, it is found that there are some shortcomings in the evaluation methods of prefabricated building, mainly:

(1) The calculation of weights is unreasonable. Subjective weighting methods primarily rely on the subjective experience of the evaluators, leading to a high degree of subjectivity in the assigned weights. On the other hand, objective weighting methods are based on the inherent properties of the indicator data, neglecting the evaluators’ subjective agency in the evaluation process.
(2) Insufficient scientific rigor in the evaluation process. Current research findings mainly rely on expert scoring methods to assign values to indicators, which can be influenced by the experts’ cognitive levels and subjective judgments. This can compromise the accuracy and consistency of indicator valuations, potentially affecting the objectivity and reliability of the evaluation results.
(3) Complexity of the calculation process. The computational processes involved in the aforementioned evaluation models are relatively complex, making it challenging to efficiently perform calculations using conventional data analysis software. This can limit the operability and practicality of the evaluation work.

#### 1.2.3. Research gaps.

The literature review indicates that scholars are increasingly focusing on the research of evaluation methods for indicators in prefabricated building. Through the analysis above, it is found that although existing research provides valuable insights, there are still research gaps that need to be addressed:

(1) Evaluation object. Existing evaluation research primarily focuses on the overall assessment of prefabricated buildings. In contrast, evaluating the indicators for prefabricated building components offers a more detailed approach, allowing for a more precise identification of issues within prefabricated buildings. This can enhance the evaluation of collaborative optimization effects in prefabricated construction.
(2) Evaluation content. Current research on the evaluation of prefabricated buildings tends to concentrate on single indicators related to quality and construction risk, without considering the varying degrees of importance of different evaluation indicators across different stages of the lifecycle. Moreover, single indicator evaluations do not reflect the complex interrelationships among various objectives in actual construction projects, neglecting the interactions and interdependencies among the evaluation indicators.
(3) Evaluation methods. Existing evaluation methods assess the importance of the obtained indicators, yet the scientific rigor of the evaluation process is insufficient. The weighting results may lean toward subjective or objective biases, affecting the accuracy of the evaluation results.

### 1.3. Aims and originality

Prefabricated building involves various evaluation indicators throughout its lifecycle, such as duration, quality, cost, safety, and green energy. The evaluation indicator system is a complex system, with intricate interrelationships among its internal indicators. Researchers have begun to use methods from systems science and complex networks to study engineering quality and its management [30, 31]. In the field of construction engineering, Guo et al have drawn and analyzed the behavioral risk chain network of construction accidents, deriving the interactions among unsafe behaviors in construction accidents [32]. Guo et al also conducted a comparative analysis of the risk chain of accident behaviors in construction and urban rail transit construction in China using complex networks [33]. Zhou et al proposed a new method, the directed weighted accident causality network, using accident events from the UK Railway Accident Investigation Branch, to help identify potential patterns of accidents and characteristics of fault propagation [34]. Li et al applied social network analysis to identify and investigate potential networks of risk factors related to stakeholders in prefabricated residential construction projects, generating critical risks that affect progress and their interactions within the network [35]. In conclusion, the complex network theory is widely used in the research of construction project management, but mostly focuses on the study of single evaluation indicators such as quality and safety. However, in engineering practice, different construction needs correspond to different types of evaluation indicators, lifecycle impacts, and varying degrees of importance. It also overlooks the relationships between evaluation indicators, for example: emergency projects emphasize schedule indicators, often neglecting cost control indicators; safety accidents in construction are caused by factors related to building quality, and so on.

Based on the establishment of a complex network model, analyzing the importance of evaluation indicators is a typical multi-attribute decision-making problem [36]. The technique for order of preference by similarity to ideal solution (topsis) is an ideal model for multi-attribute decision-making, which can quantify qualitative concepts and evaluate the pros and cons of the objects to be evaluated [37]. Shi et al evaluated the implementation potential of prefabricated rural houses using the topsis model, and provided political, economic, social, and technological recommendations for prefabricated rural housing in Chongqing [38]. Balasbaneh et al used the topsis method to conduct a lifecycle sustainability assessment of different concrete construction technologies, thereby studying the characteristics of various concrete technologies [39]. However, research has found that the traditional topsis method typically assumes that the indicators are independent of each other, ignoring the correlation between indicators. In actual engineering practice, there is a certain correlation between evaluation indicators. The entropy weight optimized topsis method can consider the correlation between indicators, making the evaluation results more accurate and reliable [40]. At the same time, the method can more accurately reflect the information and importance between indicators and provide more stable evaluation results, reducing the variability of the evaluation results and enhancing the credibility of the evaluation.

Based on this, this paper proposes an importance evaluation method for assessment indicators that combines a complex network model with an improved topsis method, aimed at conducting importance analysis of evaluation indicators for prefabricated components across their entire lifecycle. First, relevant standards, literature, and engineering experience are utilized to identify factors influencing the connections of prefabricated building components and to clarify the relationships among these factors, thereby constructing a multi-layer complex network model. Next, complex network theory is applied to compute and analyze the importance evaluation indicators for the model nodes. Finally, the entropy-weighted optimized topsis method is employed to evaluate the nodes, selecting key nodes based on the comprehensive importance evaluation values. Simulation verification is conducted using attack nodes, and corresponding recommendations are provided for the key indicators of prefabricated components. The innovations of this research lie in:

(1) A new importance evaluation method for assessment indicators has been proposed, combining a complex network model with an improved topsis method. This paper innovatively integrates the complex network model with the entropy-weighted optimized topsis method to establish a novel framework for evaluating the importance of indicators. This approach not only comprehensively considers the interactions among multidimensional indicators such as safety, quality, and green sustainability, but also dynamically analyzes the correlations among indicators through the topological characteristics of the complex network model. Furthermore, it quantifies the importance of indicators using the improved topsis method. This methodology avoids the limitations of traditional single-indicator evaluations, enhancing the accuracy of the assessment results and the scientific basis for decision-making.
(2) A multi-level comprehensive evaluation system focused on prefabricated components has been established. This study targets prefabricated components as the evaluation object, thereby avoiding the limitations of traditional research that focuses on either the entire building or a single stage. By incorporating all lifecycle stages of components, including design, production, transportation, and construction, along with multidimensional indicators such as safety, quality, and green sustainability, a multi-level, multidimensional comprehensive evaluation framework has been constructed. This perspective enables a more precise identification of issues within prefabricated buildings, providing theoretical support for the refined management of prefabricated construction.
(3) The dynamics and vulnerability of the prefabricated component connection system have been revealed. This study analyzes the dynamic changes of the multi-layer complex network model by simulating scenarios where key nodes are attacked, uncovering the vulnerabilities and robustness of the prefabricated component connection system. This research not only identifies the key nodes that influence system performance but also provides a scientific basis for risk prevention and control as well as optimization design in engineering practice, demonstrating significant theoretical value and practical significance.

## 2. Evaluation method of prefabricated components

The multi-layer complex network model is a mathematical framework used to describe the interactions among multiple levels and factors within complex systems, containing a large number of interrelated elements. The nonlinear interactions among these elements make it difficult to comprehensively assess system behavior using a single method. The topsis model, as a classic multi-attribute decision-making method, can integrate multiple evaluation indicators and compute comprehensive scores for each node, thereby assessing the importance of the nodes [41]. However, the topsis model is unable to effectively capture the interactions between different levels and factors, and relying solely on the topsis model makes it challenging to fully reflect the complexity and dynamics of the system [40]. Therefore, this paper proposes an evaluation method that combines the multi-layer complex network model with the improved topsis method, aiming to integrate the advantages of both approaches to comprehensively consider the interactions among multiple levels and factors, thereby achieving a more accurate assessment of the system’s performance and effectiveness. The specific steps are as follows:

(1) Define the scope of the research. By clarifying the evaluation object, evaluation stages, and evaluation indicators, the scope of the research is delineated, providing a clear theoretical framework for the subsequent model construction and evaluation.
(2) Construct the multi-layer complex network model. Based on the actual project conditions, identify the evaluation indicators and build a multi-layer complex network model according to the relationships among the factors. By calculating topological indicators such as node degree, betweenness centrality, and clustering coefficient, analyze the structural characteristics of the network and quantify the importance of each node.
(3) Improve the topsis evaluation method. Optimize the topsis model using the entropy weight method to calculate the comprehensive importance evaluation values for each node, and rank the nodes based on these evaluation values to identify key nodes and critical factors.

The multi-layer complex network model constructed in this paper aims to investigate the impact of quality, safety, and green sustainability indicators on the connections of prefabricated components during the design, production, transportation, and construction phases. The focus is on analyzing the interactions among the indicators and their mechanisms affecting connection performance, rather than being limited to standardized components or large-scale production scenarios. Therefore, this model is also applicable to non-standardized components or small-batch production schemes. The multi-layer complex network model established in this study can flexibly adapt to the evaluation needs under different production scales and technical conditions, providing a scientific basis for the performance assessment of various prefabricated component connections.

### 2.1. Research scope

**Fig 1:**
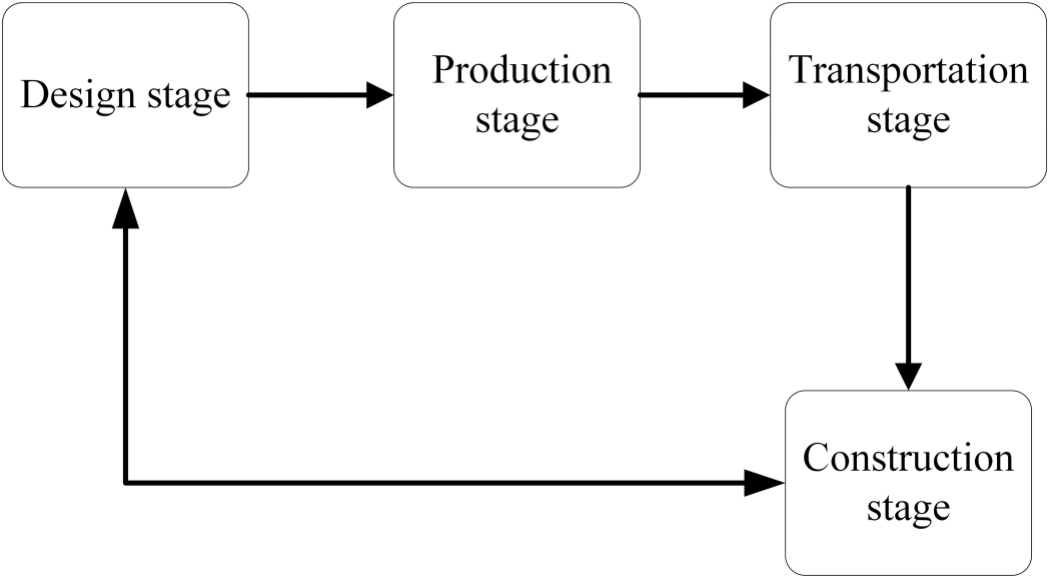
Relationship diagram of prefabricated building stages.

In this paper, the evaluation of prefabricated components’ evaluation indicators is conducted in conjunction with the whole life cycle theory. The entire life cycle mainly consists of four stages: the design stage, production stage, transportation stage, and construction stage [42], as depicted in Fig 1.

From Fig 1, it can be observed that the relationships between the various stages are interdependent and interconnected. In the design phase, the connections of prefabricated components must consider the structural performance of the nodes, the degree of design standardization, and the coordination with other components. This phase is characterized by a focus on standardization and modularization, emphasizing the reliability and constructability of the connection nodes. Therefore, the criteria for selecting evaluation indicators prioritize the rationality of node design, cost-effectiveness, and adaptability to subsequent stages [43]. In the production phase, the manufacturing of prefabricated components is influenced by mold accuracy, process parameters, quality inspection standards, and production techniques, with a primary focus on precision and quality control. This phase is highly reliant on production processes and material properties, leading to the selection criteria for evaluation indicators that emphasize manufacturing accuracy, material strength, and durability [44]. In the transportation phase, the focus of transporting prefabricated components is on safety and the protection of the nodes. This phase is characterized by the need to address complex external environments and transportation conditions. The criteria for selecting evaluation indicators emphasize the vibration resistance of the nodes, the completeness of protective measures, and transportation efficiency [45]. In the construction phase, the installation of prefabricated components must consider hoisting positioning accuracy, the stability of temporary supports, connection processes, and construction safety risks. The core focus here is on construction accuracy and structural integrity. This phase is marked by complex on-site working conditions and high technical requirements for construction. Therefore, the criteria for selecting evaluation indicators prioritize the installation accuracy of the nodes, structural integrity, and the safety of the construction process [46].

### 2.2. Construction of multilayer complex network models

Based on the definition of the research scope and the analysis of the characteristics and influencing factors of prefabricated building components throughout their entire lifecycle, this section will further construct a multi-layer complex network model to systematically describe and quantify the interactions among various evaluation indicators. Specifically, the process involves first organizing the evaluation indicators for prefabricated components to clarify their meanings and interrelationships. Second, a multi-layer complex network model will be constructed based on the associations among the evaluation indicators, depicting the node attributes and connection relationships of each indicator within the network. Finally, topological evaluation indicators such as node degree, betweenness centrality, and clustering coefficient will be selected, and the improved topsis method will be introduced to support the subsequent evaluation of node importance. The aim of this section is to provide a systematic and visual analytical framework for the comprehensive evaluation of prefabricated components, laying the groundwork for identifying key indicators and formulating recommendations.

#### 2.2.1. Evaluation index selection for prefabricated components.

For the selection of evaluation indicators for prefabricated components, this paper reviews relevant literature regarding project duration, cost, quality, safety, and green sustainability in construction engineering. A comparative analysis of the meanings and quantitative evaluation methods of these indicators is conducted. Among these, the cost and duration indicators have established quantification methods and evaluation standards in engineering practice, making their research relatively comprehensive and easy to implement. Therefore, this study focuses on addressing the evaluation issues of quality, safety, and green sustainability—indicators that are challenging to quantify yet hold significant importance. These indicators have profound implications for the long-term performance, social benefits, and environmental impact of prefabricated buildings. Specifically, the quality indicators primarily include node reliability, component accuracy, material performance, and construction quality control. The safety indicators refer to the safety of design and production, transportation and installation safety, and construction safety management. The green sustainability indicators encompass resource efficiency and environmental impact, material durability and recyclability, as well as greening of construction and production processes. By focusing on quality, safety, and green sustainability, this paper aims to provide a more scientific and comprehensive theoretical basis for the lifecycle evaluation of prefabricated component connections.

This paper takes a lifecycle approach to construction, extracting relevant evaluation indicators for quality, safety, green sustainability, and prefabricated building connections based on literature and industry standards, such as the “Green Building Evaluation Standards” [47]. Subsequently, duplicate indicators from the extracted list are removed, and indicators with similar meanings are merged, leading to a preliminary construction of the evaluation indicator system for prefabricated buildings. Building on this foundation, further screening of evaluation indicators that do not align with actual construction practices is conducted by analyzing the relationship chains of accident cases, resulting in the final evaluation indicators for prefabricated building components, as shown in Appendix A.

Appendix A includes three evaluation indicators: quality, safety, green sustainability, and the main issues related to the connection of parts and components. Q1-Q9, S1-S5, G1-G8 belong to the design phase. Q10-Q17, S6-S10, G9 belong to the production phase. Q18-Q22, S11-S15 belong to the transportation phase of the components. Q23-Q42, S16-S36, G10-G12 belong to the construction phase. In addition, standard components refer to those components with uniform parameters, such as dimensions and reinforcement, which facilitate mass production in component manufacturing plants.

#### 2.2.2. Modeling the network.

In the multi-layer complex network model, nodes represent the fundamental units of the network, corresponding to specific indicators or influencing factors within the prefabricated component evaluation system. For example, quality indicators, safety indicators, and green sustainability indicators can all serve as nodes within the network. Each node not only encompasses the specific attributes of the indicator but also connects to other nodes through edges, reflecting the interactions among the indicators. The definition of nodes is based on the actual connotations of the evaluation indicators and their roles throughout the lifecycle of prefabricated buildings, ensuring that the model accurately characterizes the features of each indicator and its impact on the overall performance of the system.

The hierarchical division of the model is based on the phased nature of the lifecycle of prefabricated components and the categorical classification of evaluation indicators. First, the nodes are categorized into three main categories—quality, safety, and green sustainability—according to the nature of the evaluation indicators, with each category forming a sub-layer of the network. Second, within each sub-layer, the indicators are further associated with the four phases of design, production, transportation, and construction, resulting in a multi-level, multidimensional network structure. Finally, by systematically analyzing the logical relationships among the indicators, the connections (edges) between nodes and their weights are determined, integrating the multi-level, multidimensional network structure into a unified comprehensive hierarchy. This approach constructs an overall network model that can comprehensively reflect the intrinsic relationships within the prefabricated component evaluation system. This hierarchical division not only embodies the systematic nature of the evaluation framework but also clearly illustrates the roles of each indicator at different stages and their interrelationships.

The physical significance of the multi-layer complex network model lies in its ability to comprehensively and dynamically reflect the interactions among various indicators within the prefabricated component evaluation system and their impact on the overall system performance. First, the model, through its multi-level and multidimensional network structure, can integrate the interactions of indicators such as quality, safety, and green sustainability, thereby avoiding the limitations of single-indicator evaluations. Second, by simulating scenarios where key nodes are attacked, the model can reveal the vulnerabilities and robustness of the system, providing theoretical support for risk prevention and control. Finally, the model visually presents the relationships among indicators through the topological structure of nodes and edges, offering decision-makers a clear analytical perspective. The multi-layer complex network model not only embodies the scientific nature of the model but also provides a theoretical basis for its application in engineering practice.

Many researchers have proposed different methods to establish network models, but the construction of the network typically impacts the analysis results. In order to avoid the influence of subjective factors on the analysis results, we determine the relationships between evaluation indicators by extracting the relationship chains of accident cases. According to accident reports, the factors leading to various indicators are not isolated but interconnected. This implies the existence of information propagation, energy exchange, interaction, or other forms of interplay among these factors. The accident cases involved in this paper mainly come from the “Analysis of Construction Safety Accidents [48]” organized by the Engineering Quality and Safety Supervision Department of the Ministry of Housing and Urban-Rural Development, and reference other standards and regulations such as “Technical Specification for Safety of High-altitude Operations in Construction of Buildings” (JGJ 80–2016) [49], “Code for Construction and Acceptance of Crane Installation Engineering” (GB 50278–2010) [50], and “Classification of Injury and Accident of Enterprise Employees” (GB 6441–1986) [51], etc. The process of identifying relationships between evaluation indicators is shown in Table 1.

**Table 1:** Example of a recognized event chain.

Accident Name	Yichuan County, Henan Province 3.1	
Accident Process	When all maintenance personnel were standing in the hoist cage to carry out maintenance work while the hoist control malfunctioned, causing the hoist cage to continue to ascend and collide with the top. The wire rope on the upper linkage pulley of the hoist cage was dislodged from the fixed clamp plate due to the disengagement of the pulley pin from the clamp plate pin hole, causing the wire rope and pulley to detach from the fixed clamp plate. As a result, the hoist cage lost lifting force and fell freely from a height of approximately 10 meters to the ground, leading to the fall of the maintenance personnel and others who were on the hoist cage at the time.	
Accident Cause	Immediate causes: Maintenance personnel violated regulations; the involved elevator itself had defects; the control of the elevator suddenly malfunctioned, and the safety protection device did not function; the operator did not receive safety training; managerial negligence. Indirect causes: The construction unit and the construction party did not strictly adhere to effective construction management procedures; the construction party had disorganized pre-construction organization and management; the county housing department did not rigorously review and supervise the filing of the winning bid notice and the issuance of the construction permit.	
Relationship Chain	The lax issuance of construction permits by the housing department → the construction unit’s failure to strictly adhere to construction procedures → disorganized on-site organization and management	The lifting and installation personnel did not receive safety training and education for construction → managerial staff had limited work experience → issues with the construction machinery → poor level of construction machinery → substandard construction.
Code	Q42→Q41→S27	S16→S21→Q40→G10→L3.

**Fig 2:**
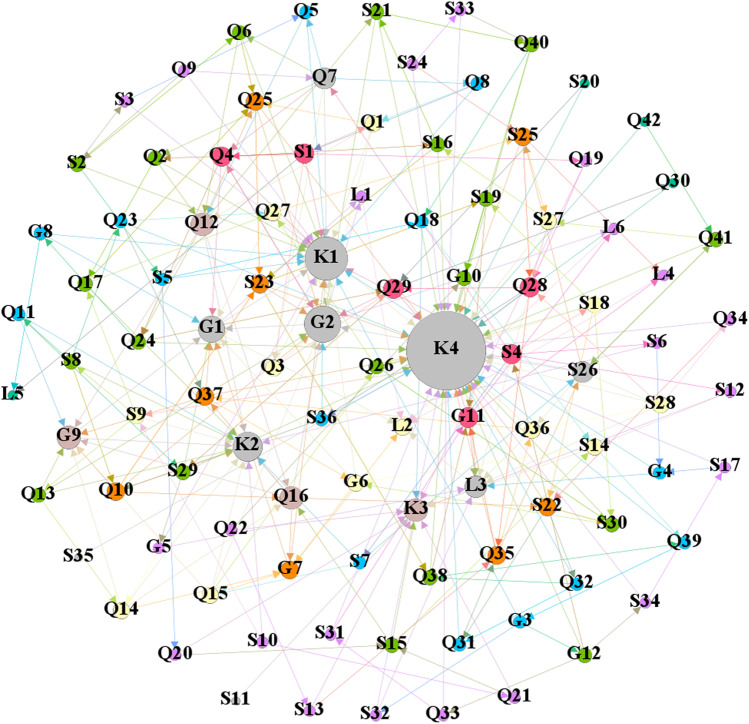
Model of evaluation index network of prefabricated components.

The first line in Table 1 represents the names of the accidents, such as the “3.1 Incident in Yichuan County, Henan Province.” The second line provides a brief description and key information about the accidents. The third line lists the direct and root causes of the accidents as identified in the accident reports. In the fourth line, the event chain is condensed from the accident report. The direction of the edges between nodes in the network can be derived from the relationships between the various factors. Finally, event chains are represented using symbols, as shown in the fifth line. Similar methods are used to identify the relationships between other indicator factors, with all the event chains from the reports listed in Appendix B. Based on the identification of all event chains, we used the Gephi software to construct a complex network model, as shown in Fig 2.

#### 2.2.3. Indicators for evaluating the importance of model nodes.

This paper employs complex network methods to calculate the topological properties of the model. Different indicators are utilized to varying extents across different network structures, and a single indicator cannot comprehensively reflect the importance of all nodes within the network [52]. Therefore, to measure the importance of evaluation indicators for prefabricated buildings, this study selects several importance evaluation indicators, including node degree, betweenness centrality, closeness centrality, clustering coefficient, and eccentricity. Consequently, these nodes do not include the four key phase nodes and the six nodes related to connection issues of prefabricated components.

(1) Degree centrality

Degree is the most direct measure of centrality in network analysis, and a node’s degree reflects its importance in the network. In a directed network, a node’s degree (*k*_*i*_) includes both out-degree and in-degree. Out-degree ($k_{i}^{out}$ ) refers to the number of edges from node *i* to other nodes, while in-degree ($k_{i}^{\text{in}}$) refers to the number of edges from other nodes pointing to node *i*. The out-degree, in-degree and degree of node *i* are calculated as shown in equations (1)–(3).


$$k_{i}^{out}=\sum_{j=1}^{N}a_{ij}$$ (1)

$$k_{i}^{in}=\sum_{j=1}^{N}a_{ji}$$ (2)

$$k_{i}=k_{i}^{in}+k_{i}^{out}$$ (3)

Where *N* is the number of nodes in the network, *a*_*ij*_ represents the edge from node *i* to node *j*, and *a*_*ij*_ represents the edge from node *j* to node i.

(2) Betweenness centrality

Betweenness centrality is a measure of node importance based on the number of shortest paths that pass through a certain node, reflecting the node’s intermediary capacity and its significant role in information transmission between nodes. A higher betweenness centrality indicates a more important position of the node in the network. Its definition is given by equation (4):


$$R_{BC_{i}}=\sum_{s\neq i\neq t}\frac{n_{st}^{\left(i\right)}}{g_{st}}$$ (4)

Where: $n_{st}^{\left(i\right)}$ represents the number of shortest paths between nodes *s* and *t* that pass through node *i*; $g_{st}$ represents the total number of shortest paths between nodes *s* and *t*.

(3) Closeness centrality

Closeness centrality is an indicator that measures the proximity of a particular node to other nodes within a network. It reflects the central position of the node in the network, that is, the average distance between the node and all other nodes. A higher closeness centrality indicates a shorter distance to other nodes, which implies greater efficiency in information dissemination or influence propagation. Its definition is given by equation (5):


$$C_{i}=\frac{N-1}{\sum_{i\neq j}d_{ij}}$$ (5)

Where: $C_{i}$ represents the closeness centrality of node *i*, *N* is the total number of nodes in the network, $d_{ij}$ is the shortest path length between node *i* and node *j*, and $\sum_{i\neq j}d_{ij}$ is the sum of the shortest path lengths from node i to all other nodes in the network.

(4) Clustering coefficient

The clustering coefficient describes the level of connections between neighboring nodes of a node, representing the network’s node-to-node closeness or clustering. The value of the clustering coefficient ranges from 0 to 1. A clustering coefficient close to 1 indicates a high level of clustering, and vice versa. The definition is shown in equation (6).


$$C_{i}=\frac{E_{i}}{k_{i}\left(k_{i}-1\right)}$$ (6)

Where: *E*_*i*_ refers to the number of edges between *k*_*i*_ neighboring nodes of node *i*.

(5) Eccentricity

Eccentricity refers to the shortest longest path length between a node and all other nodes in the network. It indicates the distance or closeness of a node to other nodes. The definition is shown in equation (7).


$$E_{cc}=\max\left(d\left(k,k_{i}\right)\right)$$ (7)

Where: *k* represents the node for which eccentricity is being calculated, *k*_*i*_ represents other nodes in the network, and *d (k, k*_*i*_*)* represents the shortest path length from node *k* to node *k*_*i*_.

### 2.3. Entropy weight optimization topsis model

Based on the constructed multi-layer complex network model and the selected importance evaluation indicators, this section will further employ the improved topsis method for a comprehensive quantitative analysis of the evaluation indicators for prefabricated components. The topsis model, as a commonly used multi-attribute decision-making method, when combined with the multi-layer complex network model that describes the interactions among multiple levels and factors within complex systems, can adequately consider the interactions among various levels and factors, thereby enhancing the accuracy and reliability of decision-making [53]. However, traditional topsis methods exhibit subjectivity in weight allocation, making it difficult to fully reflect the intrinsic relationships among indicators [6]. Therefore, this paper introduces the entropy weight method to optimize the topsis approach, enhancing the scientific rigor and accuracy of the evaluation results through objective weight assignment. The steps are outlined as follows:

(1) Dimensionless processing

Standardize the network importance evaluation indicators for all prefabricated component factors. Node degree, betweenness centrality, closeness centrality, and clustering coefficient are considered positive indicators, and the smaller the eccentricity, the better, as it is a negative indicator. These are calculated using formulas (8) and (9) respectively. *X*_*ij*_ represents the *j*-th important evaluation indicator of the *i*-th factor, *m*_*j*_ represents the minimum value of the evaluation indicator, and *M*_*j*_ represents the maximum value of the evaluation indicator.


$$X_{\text{ij}}=\left(x_{ij}-m_{j}\right)/\left(M_{j}-m_{j}\right)$$ (8)

$$X_{\text{ij}}=\left(M_{\text{ij}}-x_{ij}\right)/\left(M_{j}-m_{j}\right)$$ (9)

(2) Zeroing processing

Since the minimum value of the processed evaluation data is 0, it is necessary to perform a positive shift to eliminate zero values. All data will be uniformly increased by a small value *α* to ensure meaningful data operations. In this study, *α* = 0.0001 is used, and the calculations are performed according to equation (10).


$$X=X_{ij}+\alpha$$ (10)

(3) Entropy weight calculation

Calculate the characteristic proportion *P*_*ij*_ of the indicator value of each factor to the sum of all factor indicator values, as shown in equation (11). *P*_*ij*_ represents the probability of the *i*-th influencing indicator under the *j*-th important evaluation indicator, with m being the number of influencing factors.


$$P_{ij}=X_{ij}/\sum_{i=1}^{m}X_{ij}$$ (11)

Calculate the entropy of each indicator (*e*_*j*_) using formula (12),


$$e_{j}=-\sum_{i=1}^{n}P_{ij}\ln P_{ij}\ln m,0\leq e_{j}\leq 1$$ (12)

Calculate the coefficient of variation (*g*_*j*_) for each indicator using formula (13).


$$g_{j}=1-e_{j}$$ (13)

Subsequently, calculate the entropy weight (*W*_*j*_) for each indicator using formula (14), where m represents the number of evaluation indicators.


$$W_{\text{j}}=g_{j}/\sum_{i=1}^{m}g_{j},j=1,2,3\cdots ,m$$ (14)

(4) Determine the optimal vector (*Z*^*+*^) and the worst vector (*Z*^*-*^) of the matrix (*Z*).

Utilize the calculated evaluation indicator entropy weights to construct a normalized weighted matrix and compute the *Z*^*+*^ and *Z*^*-*^ for each evaluation object, as shown in equations (15) and (16).


$$Z^{+}=\left(Z_{1}^{+}�Z_{2}^{+}�\cdots �Z_{\text{m}}^{+}\right)�Z_{\text{j}}^{+}=\max\left(Z_{1j}^{+}�Z_{2j}^{+}�\cdots �Z_{\text{mj}}^{+}\right)$$ (15)

$$Z^{-}=\left(Z_{1}^{-}�Z_{2}^{-}�\cdots �Z_{\text{m}}^{-}\right)�Z_{\text{j}}^{-}=\max\left(Z_{1j}^{-}�Z_{2j}^{-}�\cdots �Z_{\text{mj}}^{-}\right)$$ (16)

(5) Calculate the relative closeness

By computing the Z^+^ and Z^-^ of the weighted decision matrix, calculate the positive ideal solution (*D*_*i*_^*+*^) and negative ideal solution (*D*_*i*_^*-*^) using the Euclidean distance formula (17). Then, determine the relative closeness (*A*_*i*_) of the optimal solution using formula (18). A higher relative closeness indicates better evaluation quality, ultimately determining the comprehensive ranking of the superiority and inferiority.


$$D_{i}^{+}={\left[\sum_{j=1}^{m}W_{j}{\left(Z_{ij}-Z_{j}^{+}\right)}^{2}\right]}^{1/2}�i=1,2,\cdots ,n$$ (17)

$$D_{i}^{-}={\left[\sum_{j=1}^{m}W_{j}{\left(Z_{ij}-Z_{j}^{-}\right)}^{2}\right]}^{1/2}�i=1,2,\cdots ,n$$ (18)

## 3. Case study

The multi-layer complex network model established above covers the common control factor nodes of safety, quality, and green sustainability in the assembly of building components. Such a model is helpful in identifying and analyzing various control factors that may affect the final outcome of assembly building projects comprehensively. However, it should be noted that each specific engineering project has its unique characteristics and requirements. In practical applications, it is neither possible nor necessary to consider all potential influencing factors. Therefore, the method proposed in this paper needs to be appropriately identified and adjusted according to the actual situation of specific projects in order to ensure the applicability and effectiveness of the model.

### 3.1. Case background

**Fig 3:**
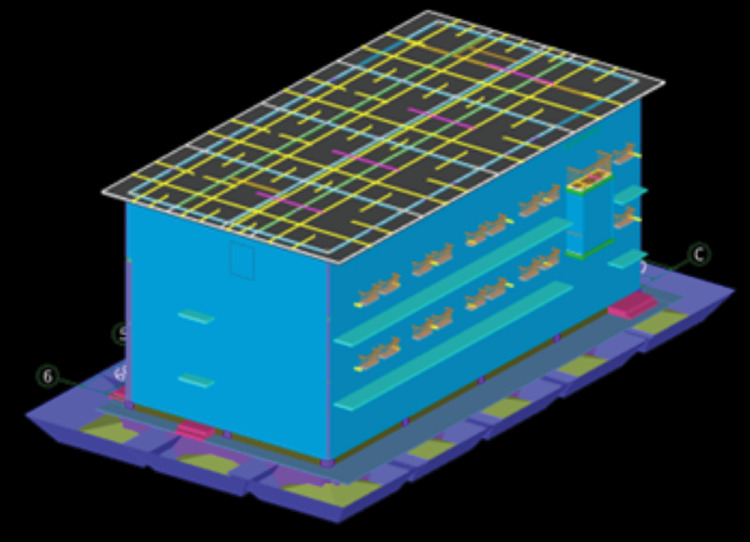
Architectural model of construction projects.

**Fig 4:**
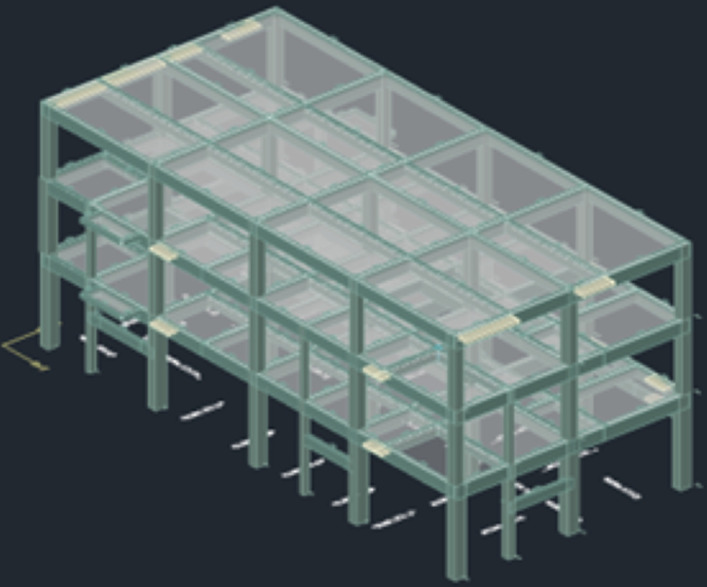
Structural model of construction projects.

The project is located in Shenzhen, Guangdong Province, featuring a frame structure with a building area of 1054.86 square meters and three above-ground levels. The project involves the extensive use of prefabricated components, including frame lines, frame beams, composite floor slabs, walls, and staircases. By utilizing the Guanglian BIM Civil Engineering Measurement Platform GTJ2021 and PKPM-PC for three-dimensional modeling, the project’s scenario is presented in Fig 3 and 4. The accident of this project is briefly described as follows: The project started in July 2019. During the transportation of prefabricated components, the factory did not provide adequate safety protection, resulting in damage to the components. However, the construction site did not conduct acceptance inspection and randomly piled the damaged components. In the process of formwork erection, the workers without special operation certificates carried out the work without specific plans and technical disclosure. The actual person in charge of the construction unit did not organize personnel for overall acceptance inspection in accordance with regulations and entered the next stage without completing signature procedures. The supervision unit did not issue a supervision notice or stop-work order, allowing the construction site to proceed to the next stage. During the construction process, maintenance personnel stood on the hoist cage to conduct maintenance on the hoist gantry without taking safety measures. As a result, the hoist malfunctioned and caused an injury accident. In addition, there were violations of regulations in the use of other mechanical equipment such as component lifting. Based on the complete construction process and accident records of the project, key data covering the design, production, transportation, and construction phases have been included, ensuring the representativeness of the data and the reliability of the model.

### 3.2. Construction of project-specific multilayer complex network models

#### 3.2.1. Model establishment approach.

**Fig 5:**
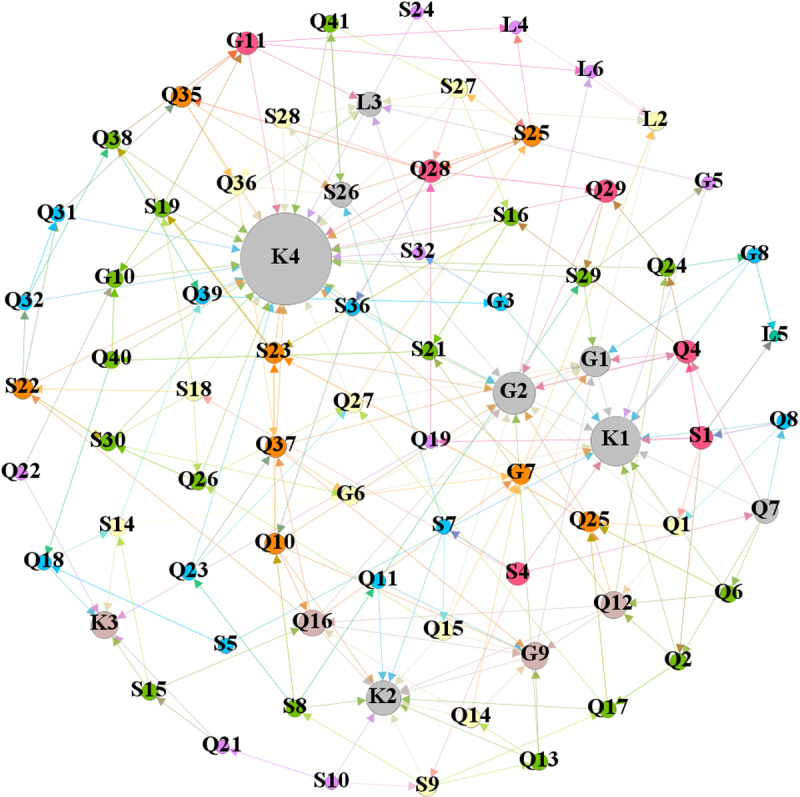
Analysis process of the specific project.

Based on the complete construction process and accident records of a specific project, a multi-layer complex model combined with the improved TOPSIS method is employed for the analysis of the particular prefabricated construction project. Through a standardized approach to model processing and data handling procedures, the scientific rigor and practicality of the research conclusions are effectively ensured. The specific steps are illustrated in Fig 5.

A project-specific multilayer complex network model was constructed based on the analytical process in Fig 5. Firstly, information about the project and relevant accident situations is collected, and a detailed analysis of the construction process and accident information is conducted. Based on Appendix A, safety, quality, and green sustainability issues present in the project are identified. Secondly, according to the relationship identification results of the indicators in Appendix B, the subsequent level of nodes influenced by these nodes is clarified, determining the nodes and their connections in the multi-layer complex network model. Unidentified nodes indicate that such factors are absent in the engineering practice project. The greater the number of unidentified nodes, the fewer issues related to safety, quality, and green sustainability that contribute to problems in the connections of prefabricated components in the project. Next, important evaluation indicators are selected, and topological indicators such as node degree and betweenness centrality are calculated to quantify the importance of the nodes in the project. Finally, the nodes of the project are evaluated using the entropy-weighted optimized topsis method, and the critical nodes of the project are identified based on the comprehensive importance evaluation values.

#### 3.2.2. Model establishment.

By identifying the safety, quality, and green sustainability factors of the project, it was found that the identified factors correspond entirely to the factors in Appendix A. Table 2 shows the safety, quality, and green sustainability factors identified for the project.

**Table 2:** Safety, quality, and green sustainability factors identified for the project.

Number	Type	Factor
1	Quality factors	Q1,Q2,Q4,Q6,Q7,Q8,Q10,Q11,Q12,Q13,Q14,Q15,Q16,Q17,Q18,Q19,Q21,Q22,Q23,Q24,Q25,Q26,Q27,Q28,Q29,Q31,Q32,Q35,Q36,Q37,Q38,Q39,Q40,Q41
2	Safety factors	S1,S4,S5,S7,S8,S9,S10,S14,S15,S16,S18,S19,S21,S22,S23,S24,S25,S26,S27,S28,S29,S30,S32,S36.
3	Green sustainability factors	G2,G3,G5,G6,G7,G8,G9,G10,G11
4	Problems with connection of components	L2,L3,L4,L5,L6
5	Key stage	K1,K2,K3,K4

**Fig 6:**
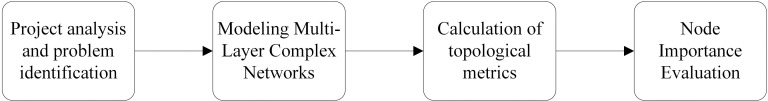
Multi-layer complex network model of the project.

Based on the identified influencing factors, the establishment of the multi-layer complex network model for this project should include the control factors of each category in Table 2 and the nodes they affect. This is mainly because issues in managing prefabricated components may lead to the occurrence of other problems. Therefore, the establishment of the multi-layer complex network model for this project is shown in Fig 6 below.

### 3.3. Results analysis

#### 3.3.1. Analysis of importance evaluation indicators.

(1) Node degree

**Fig 7:**
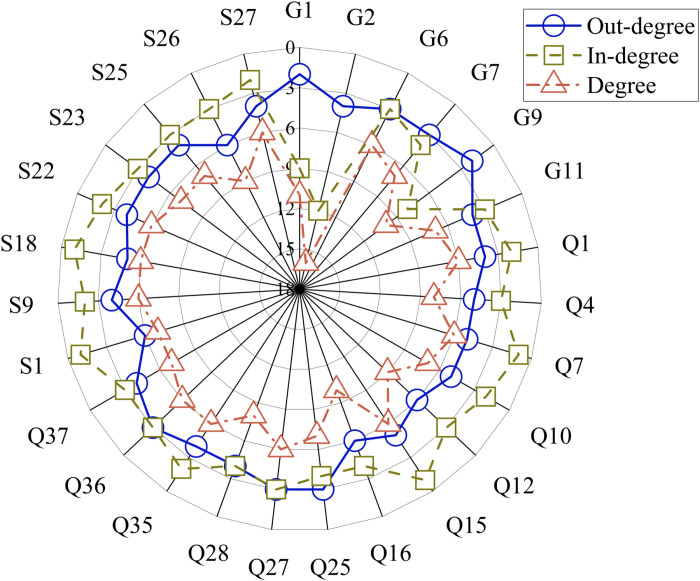
Total degree, in-degree, and out-degree of nodes with total degree greater than 5.

Using Gephi, the node degree indicators of the multi-layer complex model for this project are calculated. Nodes with higher in-degree are more susceptible to the influence of other nodes. Conversely, nodes with higher out-degree are more likely to influence other nodes. The calculation yields the out-degree, in-degree, and total degree values for nodes with a total degree greater than 5, as shown in Fig 7.

As shown in Fig 7, nodes “G2: Inadequate connection of non-structural components and equipment in buildings”, “G1: Insufficient structural capacity and building functionality”, “G9: Low production level of components”, “Q16: Inadequate component strength”, and “S26: Inadequate supervision by the supervisory unit” have relatively high total degrees, with values of 16, 11, 10, 10, and 9 respectively. These factors play important roles in the connection of prefabricated components as they can directly influence other factors or be influenced by them. “G2: Inadequate connection of non-structural components and equipment in buildings” has the highest total degree of 16, the highest in-degree of 12, and an out-degree of only 4. This indicates that this evaluation indicator may lead to inadequate connection of non-structural components and equipment in buildings due to 12 influencing factors. The factors “Q16: Inadequate component strength” and “S26: Inadequate supervision by the supervisory unit” have the highest out-degrees, with a value of 6, indicating that these evaluation indicators could lead to the occurrence of 6 factors. The average degree value of the network model is 3, indicating that on average each indicator is connected to 3 other indicators.

(2) Betweenness centrality

**Fig 8:**
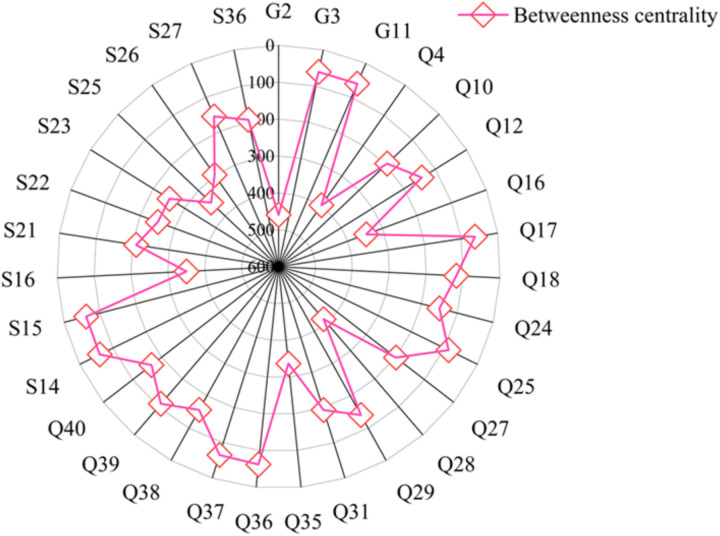
Nodes with betweenness centrality values greater than 60.

By calculating the betweenness centrality indicators, we can understand the role of the nodes in the project’s connections. After the calculation using Gephi, stages with betweenness centrality greater than 60 are obtained, as shown in Fig 8.

As shown in Fig 8, nodes “G2: Inadequate connection of non-structural components and equipment in buildings”, “Q28: Inadequate implementation of responsibilities by each party”, “Q4: Difficulties in ensuring the design quality between component nodes and gaps”, “S16: Lack of safety training and education for lifting and installation personnel”, and “S25: Unauthorized commencement of work” have relatively high betweenness centrality values, with values of 459.25, 412.7, 397.05, 350.31, and 347.82 respectively. These factors play more intermediary roles in the connection of prefabricated components, and they have a more significant impact on the transmission of information and connections within the network. The node “G2: Inadequate connection of non-structural components and equipment in buildings” has the highest betweenness centrality of 459.25, indicating its greater influence and control in transmitting information and influencing other nodes. Nodes “S4”, “S5”, “S10”, “S24”, and “Q22” have a betweenness centrality of 0, indicating that these nodes do not play intermediary roles in the network and their removal or failure has a relatively minor impact on the overall structure and information transmission of the network.

(3) Closeness centrality

**Fig 9:**
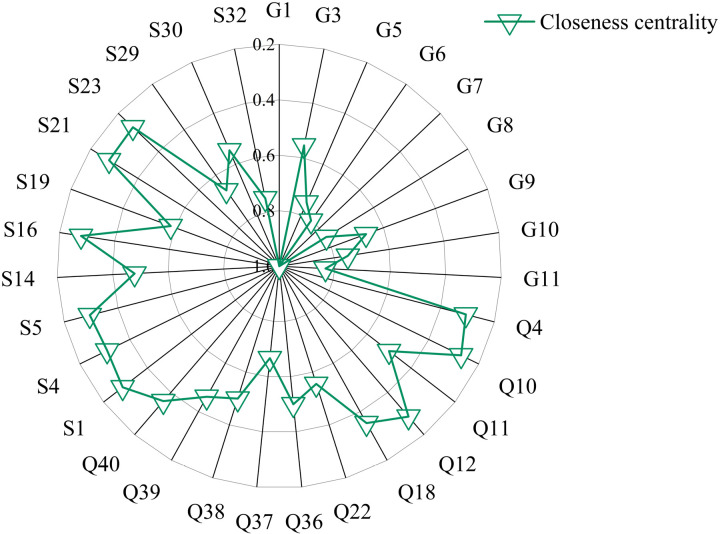
Nodes with closeness centrality values greater than 0.26.

Closeness centrality reflects the distance from a stage to other points in the network. By calculating the closeness centrality indicators for each node in the project, nodes with closeness centrality greater than 0.26 are obtained, as shown in Fig 9.

As shown in Fig 9, nodes “G1: Insufficient structural capacity and building functionality”, “G7: Poor durability and maintenance of building materials”, “G11: Low level of component lifting and installation”, “G6: Lack of measures to improve the durability of prefabricated components”, and “G8: Low standardization of component design” have relatively high closeness centrality values, with values of 1, 1, 0.84, 0.8, and 0.8 respectively. These factors have more direct neighboring nodes or shorter shortest paths to other nodes, playing important roles in connecting and disseminating information within the network. The node “G1: Insufficient structural capacity and building functionality” has the highest closeness centrality of 1, indicating that it is very close to other nodes, has more direct neighboring nodes, and can more quickly transmit information and influence other nodes, demonstrating significant importance and influence. The node “Q32: Failure to prepare a specific construction plan for guiding construction” has a closeness centrality of 0.17, indicating that it has fewer direct neighboring nodes or sparse connections to other nodes in the network, potentially indicating lower importance and influence.

(4) Clustering coefficient

**Fig 10:**
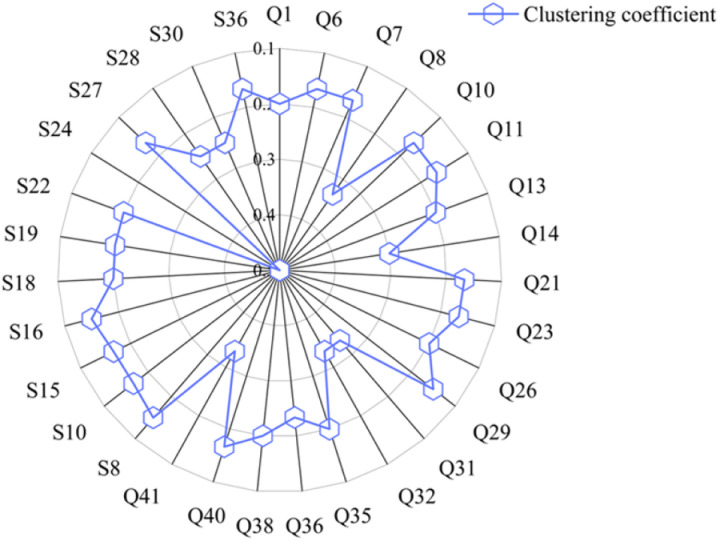
Nodes with clustering coefficient values greater than 0.15.

The clustering coefficient reflects the closeness between nodes. By calculating the clustering coefficient indicators for each node in the project, nodes with a clustering coefficient greater than 0.15 are obtained, as shown in Fig 10.

As shown in Fig 10, nodes “S24: Illegal bidding by the construction unit”, “Q31: Blind construction without following the construction plan”, “Q32: Failure to prepare a specific construction plan for guiding construction”, “Q41: Failure of the construction unit to strictly adhere to construction procedures”, and “Q8: Design plan not in compliance with standard specifications” have relatively high clustering coefficients, with values of 0.5, 0.33, 0.33, 0.3, and 0.33 respectively. This indicates that the connections between neighboring nodes of these nodes are more closely knit, meaning that the neighboring nodes of a node are more likely to be interconnected. This suggests that the local area where these nodes are located is more densely connected, containing more triangular relationships. “S24”, “Q31”, “Q32”, “Q41”, and “Q8” have the highest clustering coefficients, indicating that these nodes have higher clustering in terms of information transmission, social interaction, and functional differentiation. Nodes “S5”, “S32”, “Q19”, “Q22”, and “G3” have a clustering coefficient of 0, indicating that the local area where these nodes are located has no connections.

(5) Eccentricity

**Fig 11:**
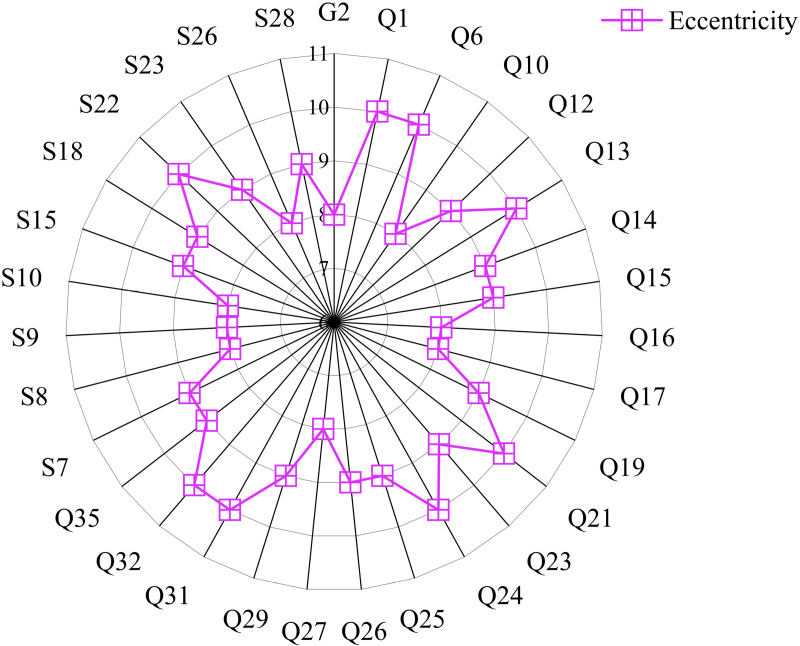
Nodes with eccentricity values greater than 7.

By calculating the eccentricity of the multi-layer complex network of this project using Gephi, we can understand the distance or closeness between nodes. Nodes with eccentricity greater than 7 are obtained, as shown in Fig 11.

As shown in Fig 11, nodes “Q1: Lack of reserved installation deviation for prefabricated components”, “Q24: Conflict between line pipe laying and truss during installation of prefabricated slabs”, “Q6: Overlapping positions of designed components, causing collision with pipelines”, “Q13: Surface crack issues”, and “S22: Insufficient bearing capacity of transport lifting machinery” have eccentricity values of 10. This indicates that these factors are relatively isolated within the network, with weaker connections to other nodes, possibly having fewer direct paths or longer paths to reach other nodes. The node “Q1: Lack of reserved installation deviation for prefabricated components” has the highest eccentricity of 10, indicating fewer connections to other nodes. The nodes “G1: Insufficient structural capacity and building functionality” and “G7: Poor durability and maintenance of building materials” have the lowest eccentricity of 1, indicating the shortest distance to other nodes and thus being the most central nodes in the network. This suggests that these nodes have the shortest paths to other nodes and are closely connected to them.

#### 3.3.2 Improved topsis evaluation.

Based on the analysis of the importance evaluation indicators of the multi-layer complex network model for this project, in order to identify the key nodes of the project, we used the Matlab.2019b platform to calculate the comprehensive importance evaluation values of various indicators for the prefabricated components of the project. These values were used to rank the importance of the indicators. Studies have shown that if 5% to 10% of the key nodes in a network fail, it can have a large impact on the network [54]. Therefore, this paper lists the top 10% nodes in terms of comprehensive evaluation importance ranking, as shown in Table 3.

**Table 3:** Importance ranking of nodes for the prefabricated components of the project (top 10%).

Node number	Comprehensive importance rating value	Importance ranking
G2	0.579	1
Q28	0.542	2
Q4	0.528	3
Q35	0.510	4
Q16	0.509	5
S25	0.493	6
S16	0.489	7
S26	0.474	8

Based on the topsis method for node evaluation, nodes G2, Q28, Q4, Q35, Q16, S25, S16, and S26 play significant roles in the network of this project and are identified as critical nodes for the prefabricated components. In terms of the distribution of these key nodes, quality, safety, and green sustainability-related factors account for 50%, 37.5%, and 12.5% of the top 10% of key nodes, respectively, indicating that quality issues are the primary driving factors behind connection problems. Regarding phase distribution, key nodes in the construction phase account for 50%, followed by the design phase, production phase, and finally the transportation phase. This suggests that the construction phase, due to inadequate personnel operations, responsibility enforcement, and process standardization, becomes a critical stage for the accumulation of risks.

### 3.4. Simulation verification

**Fig 12:**
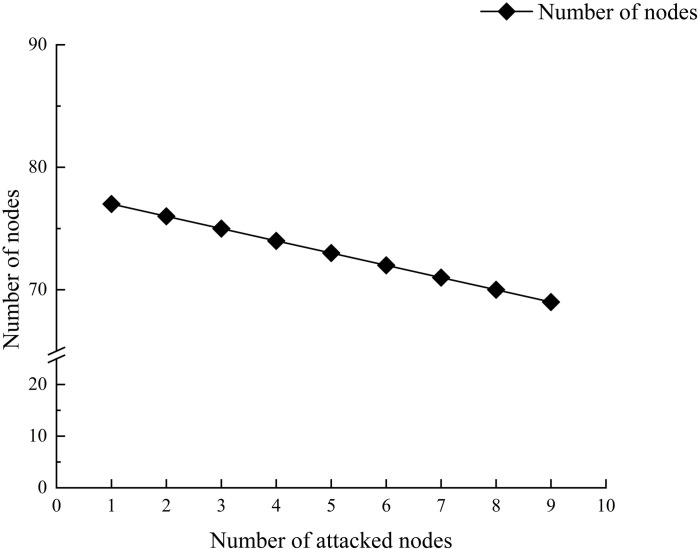
Number of nodes in the attacked complex network.

**Fig 13:**
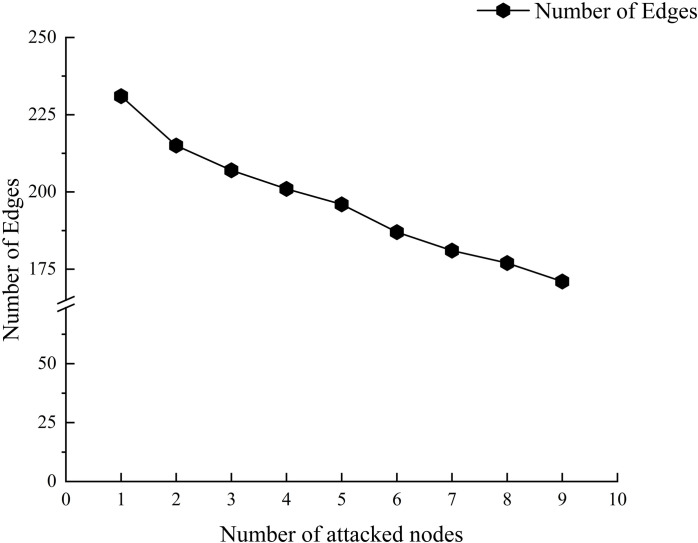
Number of edges in the attacked complex network.

**Fig 14:**
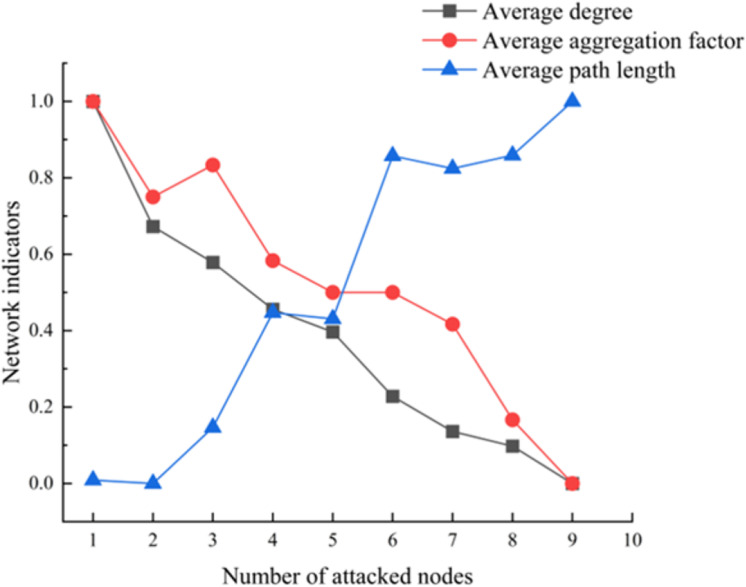
Network indicators of the attacked complex network.

The evaluation using the entropy weight-topsis model indicates that the 8 nodes play crucial roles in the prefabricated components. Avoiding issues with these 8 critical nodes at various stages throughout the entire lifecycle of prefabricated buildings can significantly reduce safety, quality, and green sustainability issues in the connection of prefabricated components. To quantitatively and qualitatively demonstrate this, we conducted simulations using Python. We sequentially attacked each critical node, from the smallest to the largest sequence number. Additionally, after each attack, we calculated the number of nodes, edges, average path length, clustering coefficient, and network density. The simulation results are shown in Figs 12–14.

Figs 12 and 13 show that after the critical nodes are attacked, both the number of nodes and edges in the multi-layer complex network decrease. Notably, the number of edges decreases rapidly from 231 to 171. This indicates that critical nodes have a high degree of connectivity within the network, and their failure leads to the rapid disappearance of the edges connected to them, thereby compromising the overall structure of the network. The swift reduction in the number of edges reflects a decline in network connectivity, suggesting that the pathways for the propagation of issues related to the connections of prefabricated components are severed, thus reducing the likelihood of problems occurring. This result reveals the central role of critical nodes in maintaining the integrity of the system from the perspective of network structure.

Fig 14 shows that as critical nodes are attacked, both the average degree and the average clustering coefficient of the network exhibit a downward trend. The decrease in the average degree indicates a reduction in the connectivity density among nodes in the network, leading to weakened interactions between them. The reduction in the clustering coefficient reflects a diminished local cohesiveness within the network, resulting in a decrease in the synergistic effects among nodes. These changes suggest that the failure of critical nodes undermines the network’s compactness and synergy, significantly reducing the range and impact of the propagation of issues related to the connections of prefabricated components. This finding highlights the crucial role of critical nodes in maintaining the coherence and robustness of the network. As critical nodes are attacked, the average path length of the network significantly increases. The rise in average path length indicates a decline in the efficiency of connections between nodes, resulting in longer paths for the transmission of information or problems. This change implies that the failure of critical nodes not only weakens the connectivity of the network but also reduces the efficiency of problem propagation within it. From the perspective of prefabricated construction, this phenomenon suggests that the failure of critical nodes can effectively isolate localized issues, preventing their spread throughout the entire system and thereby enhancing the overall stability of the system.

The simulation results of node attacks not only validate the central role of critical nodes in the connection issues of prefabricated components but also reveal the theoretical value of the multi-layer complex network model in analyzing system vulnerabilities and risk prevention. From a theoretical perspective, the identification of critical nodes and their impact mechanisms on network structure provide a new perspective for the study of robustness in complex systems. From a practical standpoint, the research findings offer a scientific basis for risk prevention and control throughout the lifecycle of prefabricated construction.

## 4. Discussion

### 4.1. Advantages of the method

This paper proposes an evaluation method for prefabricated component indicators that combines a multi-layer complex network model with an improved topsis method. Considering safety, quality, green sustainability for each type of influencing factor, key stages and connectivity issues as a node, while the relationships between nodes are represented as edges, thereby constructing a multi-layer complex network model. Based on the calculation and analysis of important evaluation indicators such as node degree and clustering coefficient, the entropy-weighted optimized topsis method is employed to evaluate the nodes and identify the critical nodes. The research in this article differs from previous studies on prefabricated buildings, which mainly focus on quality or construction risk evaluation from a single aspect. Zhang, J. et al. analyzed the factors influencing the quality of prefabricated buildings based on ISM-BN and found that the construction phase has the greatest impact on building quality, and inadequate sense of responsibility among construction personnel is the most important factor that needs to be controlled [55]. Lu et al. proposed a prefabricated building construction risk assessment model based on combined weight and catastrophe progression method. Through analysis of the model, they found that the quality and standardization degree of prefabricated components, as well as the installation workspace environment, are the most important factors [56]. The method proposed in this article can consider multiple levels and factors of prefabricated buildings, providing a more comprehensive evaluation of key factors from different categories. Moreover, as prefabricated buildings involve multiple factors in practical engineering, the method proposed in this article is more in line with practical needs. Wang, S. et al. evaluated prefabricated buildings based on four aspects: assembly rate, cost, construction period, and carbon footprint, by constructing an evaluation index system [57]. Ji et al. used the DPSIR framework to determine 14 evaluation indicators for prefabricated buildings [58]. Compared with other studies on multi-factor evaluation, the method proposed in this article can sort and integrate the relationships between various factors, making each factor specific and parallel, thus clarifying the influence paths between factors. Xia, M. et al. evaluated the comprehensive risk of prefabricated buildings by determining the weights through a questionnaire survey [59], and Liu, J. et al. evaluated the safety performance of prefabricated buildings based on expert data collected through cloud models [60]. The method proposed in this paper is based on topological calculations within a multi-layer complex network and employs the improved topsis model for evaluation. This approach relies on data and scientific analysis, ensuring a high degree of reliability and accuracy. In comparison with other studies, the innovations of this paper are primarily reflected in:

(1) A framework for evaluating the importance of indicators based on a complex network model and entropy-weighted optimized topsis method has been constructed. This approach dynamically analyzes the interrelationships among multidimensional indicators through the topological characteristics of the complex network model, and combines this with the improved topsis method for quantitative evaluation. This addresses the limitations of traditional single-indicator evaluations and enhances the accuracy of assessment results and the scientific basis for decision-making.
(2) A multi-level comprehensive evaluation system focusing on prefabricated components has been established. By integrating the entire lifecycle stages of components with multidimensional indicators, this approach overcomes the limitations of traditional research that focuses on either the entire building or a single stage, providing theoretical support for the refined management of prefabricated construction.
(3) The dynamics and vulnerabilities of the prefabricated component connection system have been revealed. By simulating scenarios where critical nodes are attacked, the dynamic changes in the multi-layer complex network model have been analyzed, and key nodes affecting system performance have been identified. This provides a scientific basis for risk prevention and control as well as optimization design in engineering practice, holding significant theoretical and practical implications.

### 4.2. Research limitations

This paper provides new ideas and methods for the analysis and management of prefabricated building evaluations through the establishment of a multi-layer complex network. However, there are still limitations that affect the generalizability of the research findings and the widespread applicability of the model:

(1) Limitations in the identification of influencing factors. This study primarily identifies influencing factors by referencing existing building codes and related literature. While this approach can construct a preliminary framework of influencing factors based on established theories and practical experiences, the identification results may not comprehensively cover all connection issues related to prefabricated components. In actual prefabricated construction projects, new influencing factors may emerge due to project-specific characteristics, regional differences, or the dynamic nature of technological development, which are not adequately incorporated in existing research. Therefore, the completeness and applicability of the current model may be limited, making it challenging to fully reflect the complex and variable realities of engineering practices.
(2) Limitations in the applicability of the model. The multi-layer complex network model established in this study is primarily based on case data from prefabricated concrete projects. Its structural characteristics, construction processes, and technical requirements differ significantly from those of other structural types, such as steel structures or timber structures. Due to the unique aspects of prefabricated buildings in terms of connection methods, mechanical properties, and construction workflows across different structural types, the existing model may not be directly applicable to prefabricated construction projects of other structural types.

### 4.3. Future research

To address the aforementioned limitations, future research can be conducted in the following two areas to further refine the research framework and enhance the scientific rigor and generalizability of the model:

(1) Expand the scope of influencing factor identification. Future studies should employ a variety of methods such as field surveys, case analyses, and expert interviews to collect data from a broader range of prefabricated construction projects, particularly those project types that have not been adequately covered in existing research. By analyzing new connection issues that arise in these projects, potential influencing factors can be identified and incorporated into the existing multi-layer complex network model. Additionally, a dynamic data updating mechanism can be implemented to capture new developments in technology and engineering practices in real time, continually enriching and refining the influencing factor system of the model, thereby enhancing its adaptability and predictive capability.
(2) Expand the applicability of the model. Future research should further explore the characteristics of prefabricated construction projects of other structural types, focusing on the key influencing factors related to the connections of components and their dynamic changes. By conducting comparative analyses of the differences in connection methods, construction processes, and performance requirements among various structural types of prefabricated buildings, unique influencing factors can be identified, and the existing model can be modified and optimized. On this basis, a more universally applicable multi-layer complex network model can be constructed to accommodate prefabricated construction projects of various structural types. This research direction not only enhances the scientific rigor and practicality of the model but also provides theoretical support and practical guidance for the diversified development of prefabricated construction technologies.

Through in-depth research in these two areas, the theoretical framework of this study can be further refined, improving the model’s applicability and accuracy, and providing more scientific and comprehensive decision support for the lifecycle management of prefabricated buildings.

## 5. Conclusions

This paper proposes an evaluation method based on a multi-layer complex network model combined with an improved topsis approach. First, relevant standards, literature, and engineering experience are utilized to identify the factors influencing the connections of prefabricated building components and clarify the relationships among these factors, leading to the construction of a multi-layer complex network model. Second, complex network theory is applied to calculate and analyze the importance evaluation indicators of the model nodes. Finally, the entropy-weighted optimized topsis method is employed to evaluate the nodes, selecting key nodes based on the comprehensive importance evaluation values, and simulation validation is conducted through node attacks. A specific model is constructed and analyzed using a case study of a building project in Shenzhen, Guangdong Province, with the following conclusions:

(1) Through the analysis of important evaluation indicators such as node degree, betweenness centrality, and closeness centrality, key nodes have been identified, including “G2: Non-structural components and equipment connections of the building are not sufficiently secure,” “G1: The building structure does not meet load-bearing capacity and functional requirements,” “S24: The construction unit illegally awarded contracts,” and “Q1: Prefabricated components do not account for installation deviations.” These critical nodes play a central role in maintaining the connectivity and synergy of the network, and their stability directly determines the robustness of the prefabricated component connection system.
(2) The comprehensive evaluation results based on the improved TOPSIS method indicate that, in terms of the distribution of key nodes for the project, quality, safety, and green sustainability-related factors account for 50%, 37.5%, and 12.5% of the top 10% of key nodes, respectively. This suggests that quality issues are the primary driving factors behind connection problems. Regarding phase distribution, key nodes in the construction phase account for 50%, followed by the design phase, the production phase, and finally the transportation phase. This indicates that the construction phase, due to inadequate personnel operations, responsibility enforcement, and process standardization, is a critical period for the emergence of connection issues in prefabricated components.
(3) As critical nodes are attacked, the topological properties of the multi-layer complex network model change accordingly. The number of nodes, number of edges, average node degree, and clustering coefficient all decrease with an increasing number of attacked nodes, indicating the important role that critical nodes play in maintaining the coherence and robustness of the network. The failure of critical nodes weakens the compactness and synergy of the network. Additionally, the average path length increases as the number of attacked nodes rises, suggesting that the failure of critical nodes can effectively isolate localized issues and prevent their spread throughout the entire system, thereby enhancing the overall stability of the system.

In summary, this study reveals the key influencing factors and their mechanisms regarding connection issues in prefabricated components through theoretical analysis and simulation validation, providing a scientific basis and theoretical support for enhancing the system stability and engineering quality of prefabricated buildings. Based on these conclusions, the following recommendations are proposed:

(1) Strengthen the dynamic monitoring and optimization of critical nodes throughout their entire lifecycle. Establish a dynamic monitoring mechanism based on the identified phases of critical nodes. In the design phase, utilize BIM technology to optimize node connection designs and enhance the standardization of components. In the production phase, introduce intelligent inspection equipment to ensure the strength and precision of components. In the construction phase, implement real-time monitoring and feedback to ensure installation quality. Through dynamic optimization across the entire process, the risk of critical node failure can be reduced, thereby enhancing the overall stability of the system.
(2) Focus on quality and responsibility management during the construction phase to establish a risk prevention and control system. It is recommended to develop a risk prevention and control system centered around quality, enhancing the preparation and approval processes for construction plans, strengthening safety training and assessment for personnel, and ensuring the enforcement of responsibilities among all parties involved. Additionally, a third-party supervision mechanism should be introduced. At the same time, utilize Internet of Things (IoT) technology for real-time monitoring of the construction process to ensure compliance with construction standards and safety, thereby reducing the occurrence of quality issues at the source.

## Appendix A. List of evaluation indicators

**Table:** 

Quality factors		
Q1 Failure to allow for installation deviations in prefabricated elements	Q15 Unsatisfactory quality of raw materials for the production of components	Q29 Lack of reliable rigid connections
Q2 Prefabricated components without pre-drilled holes	Q16 Insufficient component strength	Q30 Non-compliance with the load bearing capacity of the bottom mat
Q3 Lack of reserved reinforcement for prefabricated elements	Q17 Pipeline and other buried issues	Q31 Blind construction without following the construction program
Q4 Difficulty in ensuring the quality of design between component nodes and gaps	Q18 Unreasonable stacking of components	Q32 Failure to prepare a special construction program to guide construction
Q5 Unreasonable design of component splitting, resulting in reduced stress performance and other problems	Q19 Chiseling, thickness, and truss height not meeting requirements	Q33 Poor construction site environment
Q6 Positional overlap between design elements and collision problems with piping intersections	Q20 Unreasonable transportation routes	Q34 The construction unit exceeds the unit’s qualification to undertake the project
Q7 Poor competence and quality of designers	Q21 Poor capacity and quality of loading and unloading personnel	Q35 Problems with the sequence and manner of pouring concrete elements
Q8 Design solutions do not meet standard code criteria	Q22 Insufficient load-bearing capacity of transport cranes	Q36 Inadequate organization of construction processes
Q9 The qualification of the design unit does not meet the requirements	Q23 Inadequate vibration, holes and tendon leakage between cast-in-place slabs and prefabricated slabs	Q37 Inadequate maintenance, early mold removal
Q10 Localized honeycomb holes in concrete structures	Q24 Conflict between line pipe laying and trusses during prefabricated panel installation	Q38 No managerial control during installation
Q11 Apparent quality of components	Q25 Precast slab anchoring reinforcement cannot be installed when anchored into cast-in-place beams	Q39 Installation in place without checking and calibrating prior to installation
Q12 Problems with dimensional deviations of members	Q26 Lack of grout compaction in precast elements	Q40 Problems with construction machinery
Q13 Surface cracking problems	Q27 Problems with the quality of cast-in-place strips	Q41 Failure of construction units to strictly comply with construction procedures
Q14 Leakage of reinforcement and protective layer problems	Q28 Unreasonable erection of formwork supports	Q42 Laxity in the issuance of construction permits by the housing sector
**Safety factor**		
S1 Collaborative management of design and production - transportation - installation - operations.	S13 Measures to protect components of the transportation management process.	S25 Unauthorized construction in violation of the law.
S2 Skill level of designers.	S14 Rationalization of stacking of large prefabricated components.	S26 Inadequate supervision by supervisory units.
S3 Degree of modularity and standardization of unit design.	S15 Operational status of transportation vehicles.	S27 The organization and management of the site is chaotic.
S4 Safety education training.	S16 Safety training and education for lifting and installation personnel.	S28 Lack of implementation of the responsibilities of the main bodies.
S5 Degree of rationalization of the general layout.	S17 Use of protective equipment for lifting and installing personnel.	S29 Inadequate supervision of the construction unit by the construction unit.
S6 Safety awareness and attitude of production workers.	S18 Hoisting and installation safety personnel, quality inspectors equipped with.	S30 Unauthorized operation by construction workers.
S7 Provision of production safety officers and quality inspectors.	S19 Unstandardized lifting and installation of components.	S31 General mechanical equipment disassembly and maintenance.
S8 Qualification rate of production materials and equipment.	S20 Construction site working environment.	S32 Failure to close and cordon off hazardous areas.
S9 Operational level of production staff.	S21 Work experience as a construction manager.	S33 Frequent changes of project managers.
S10 Improvement of the work safety management system.	S22 Improvement of safety management system for lifting and installation construction.	S34 Accident Early Warning and Emergency Response.
S11 Working condition of drivers and availability of driving licenses.	S23 Operational level of lifting installers.	S35 Weather and lighting conditions at the construction site.
S12 Irregular operation of loading and unloading personnel.	S24 Illegal bidding by construction units.	S36 General construction machinery and plant safety protection.
**Green sustainability indicators**		
G1 Failure of the building structure to meet the load-bearing capacity and the function of building use	G5 Failure to adopt products or accessories with safety protection	G9 Lower level of production of components
G2 Insufficiently strong connections of non-structural elements of the building, equipment	G6 Failure to take measures to enhance the durability of building component parts	G10 Poor machinery for construction
G3 No safe and secure warning and guidance signage system	G7 Poor durability and maintenance of building materials	G11 Lower level of lifting and installation of components
G4 Failure to take protective measures to safeguard personnel	G8 Less standardization in the design of components	G12 Sites should avoid hazardous areas
**Problems with connection of parts and components**		
L1 Mismatch of molding dimensions	L3 Shoddy work	L5 Design issues
L2 Strength issues	L4 Mismatch of materials	L6 Environmental factor
**Key stage**		
K1 Design stage	K2 Production stage	K3Transportation stage
K4 Construction stage		

## Appendix B. List of relationship chain

**Table:** 

Case Title	Zhoukou City, Henan Province 2.1	Dexing City, Jiangxi Province 2.6	Longhai City, Fujian Province 4.1	Fuqing City, Fu jian Province 6.2
Event chain	S1→Q1→Q25.	S1→Q2→Q12.	S3→Q5.	Q9→Q7→Q8→Q1
Case Title	Xiangxiang City, Hunan Province 7.9	Jian City, Jiangxi Province 12.20	Yanchi City, Ningxia Province 11.20	Xinyang City, Henan Province 12.19
Event chain	S7→S26.	S9→Q10→Q16.	Q7→Q3→Q14	S8→Q11→S29.
Case Title	Wenshan City, Yunnan Province 2.9	Nanning City, Guangxi Province 3.26	Xinle City, Hebei Province 4.11	Weifang City, Shandong Province 4.30
Event chain	Q15→G9	Q16→G2	Q14→G9	S5→Q20→S15.
Case Title	Linzhi City, Xizhang Province 7.5	Cangxi City, Sichuan Province 11.18	Ziyang City, Sichuan Province 11.18	Tangshan City, Hebei Province 1.30
Event chain	G8→G1	S15→S14→Q39.	Q22→S13→Q35	Q17→Q25
Case Title	Minzhong City, Sichuan Province 8.22	Xingren City, Guizhou Province 8.25	Huanggang City, Hubei Province 9.18	Suihua City, Heilongjiang Province 10.24
Event chain	Q24→G8→L5	S4→S9→S8→Q23.	S4→S18→S22→Q31.	Q14→Q16→S22→Q32
Case Title	Dali City, Yunnan Province 5.3	Liaocheng City, Shandong Province 5.7	Changsha Hunan 3.7	Zhengjiang Yunnan 3.30
Event chain	S18→S30.	Q4→G2	Q13→G2	Q23→Q37→G9
Case Title	Yuanan City, Hubei Province 5.21	Yancheng City, Jiangsu Province 8.9	Jiangyin City, Jiangsu Province 2.22	Mudanjiang City, Heilongjiang Province 6.26
Event chain	S24→S25.	S20→Q29→Q30.	S7→Q15→Q26→S30.	S23→G2→L1.
Case Title	Ganzhou City, Gansu Province 7.2	Tongren City, Guizhou Province 8.1	Yichuan City, Hehan 3.1	Wulumuqi City, Heilongjiang Province 7.1
Event chain	S1→S9→Q17.	Q1→Q12→Q16	Q42→Q41→S27	S27→Q28.
Case Title	Nanjing City, Jiangsu Province 8.17	Wuhan City, Hubei Province 8.21	Chengdu City, Sichuan Province 10.5	Guangzhou City, Guangdong Province 7.19
Event chain	S5→S20→S15→Q16.	G12→S4	S6→G4→L3.	S2→S3.
Case Title	Taiyuan City, Shanxi Province 5.14	Longkou City, Shandong Province 7.15	Linyi City, Shandong Province 8.30	Shenyang City, Liaoning Province 9.15
Event chain	S10→Q21→S15.	S4→Q7.	Q11→G9	Q7→Q5
Case Title	Xixian City, Henan Province 2.19	Baoding City, Hebei Province 3.27	Tongcheng City, Anhui Province 3.27	Weihai City, Shandong Province 3.21
Event chain	G2→G1	Q23→Q27	Q24→Q29	Q12→G9
Case Title	Yanan City, Shanxi Province 4.29	Fujin City, Heilongjiang Province 7.3	Xixianxinqu City, Shanxi Province 3.16	Wulumuqi City, Xinjiang Province 5.30
Event chain	S16→S23→Q38.	Q16→G7	Q38→G11→L3	Q14→G7
Case Title	Zhengzhou City, Henan Province 6.12	Tianbeixin City, Xinjiang Province 11.6	Suzhou City, Jiangsu Province 11.8	Guiyang City, Guizhou Province 12.23
Event chain	G7→G1	S4→S9→S8→Q23.	S18→Q26→Q27→S25.	Q15→G2
Case Title	Shijiangzhuang City, Hebei Province 8.7	Liupanshui City, Guizhou Province 11.7	Changle City, Fujian Province 11.18	Jiangbei City, Chongqing Province 3.28
Event chain	Q10→G2	G6→G1	Q17→Q27→G1	S1→Q4→S16.
Case Title	Tianshui City, Gansu Province 2.20	Zibo City, Shandong Province 6.19	Xinxiang City, Henan Province 5.1	Suihua City, Heilongjiang Province 8.3
Event chain	S4→S6.	Q16→Q37→G1	Q29→S29→G1	S4→S12→S14.
Case Title	Ninghe City, Tianjin 6.12	Ganxinanzhou City, Guizhou Province 8.13	Yulin City, Guangxi Province 8.12	Dongwan City, Guangdong Province 6.7
Event chain	S4→S7.	S1→Q3→Q29.	S18→S19.	Q23→Q37→G9
Case Title	Shanghai City, 7.19	Weifang City, Shandong Province 10.12	Dehong City, Yunnan Province 12.22	Fenghuang City, Hunan Province 8.13
Event chain	S14→Q39.	Q12→S21→G2	Q25→G2	S2→S5.
Case Title	Hefei City, Anhui Province 5.30	Xining City, Qinghai Province 4.27	Haidian City, Beijing 3.28	Yongzhou City, Hunan Province 9.21
Event chain	Q27→Q28→Q29	S24→S25.	Q1→G2	G9→G1
Case Title	Jinggu City, Yunnan Province 2.21	Wendeng City, Shandong Province 6.06	Haidian City, Beijing 2.21	Daqing City, Heilongjiang Province 8.06
Event chain	S18→S30.	S4→S12→S14.	S26→S25→S27→L3.	Q3→G2
Case Title	Dingxi City, Gansu Province 7.04	Xiangfan City, Hubei Province 1.16	Haerbin City, Heilongjiang Province 1.04	Nanan City, Chongqing Province 1.17
Event chain	S4→S6.	S4→S18→S22→Q31.	Q29→G2→Q4→G1	S4→S7.
Case Title	Baoji City, Shanxi Province 3.13	Kaifaqu City, Tianjin City 5.13	Changsha City, Hunan Province 4.30	Xiushan City, Chongqing Province 12.04
Event chain	S18→S19.	S2→S3.	S20→Q29→Q30.	S25→L4.
Case Title	Jingzhou City, Hubei Province 12.21	Zhengzhou City, Henan Province 9.06	Nanjing City, Guangxi Province 2.12	Liaocheng City, Shandong Province 12.02
Event chain	S28→L3.	G1→L2	S22→S30→G6.	Q32→Q38→Q39→G3
Case Title	Liyang City, Jiangsu Province 8.24	Fuzhou City, Guangdong Province 9.01	Lanzhou City, Gansu Province 8.31	Zibo City, Shandong Province 9.30
Event chain	S26→Q41→S26.	Q40→G10→L3	S29→G5→L3→L2.	S19→G10.
Case Title	Baoji City, Shanxi Province 7.14	Ningde City, Fujian Province 10.30	Zibo City, Shandong Province 1.10	Dalian City, Liaoning Province 5.19
Event chain	Q13→G9	S36→Q10→S23→S19.	S28→Q28→Q35.	G12→Q33→S31→G11→L6
Case Title	Ezhou City, Hubei Province 3.22	Shangyu City, Zhejiang Province 1.07	Shijiazhuang City, Hebei Province 8.19	Haerbin City, Heilongjiang Province 7.14
Event chain	L6→L2	G3→S32→L3	G12→S34→S17→G4	S25→Q28→S36→G2→L6.
Case Title	Chaoyangqu City, Beijing Province 2.27	Hangzhou City, Zhejiang Province 2.02	Mudanjiang City, Heilongjiang Province 9.10	Wanzhou City, Chongqing Province 6.21
Event chain	Q35→Q36→Q37	Q7→Q2→Q17	S28→Q36→L3.	S24→S33→Q40→Q18.
Case Title	Wenshan City, Yunnan Province 3.17	Huzhou City, Zhejiang Province 3.22	Wenshan City, Yunnan Province 3.17	Shuangyashan City, Heilongjiang Province 4.12
Event chain	Q5→Q12→Q24	S1→Q19→Q28.	Q12→Q25→S23	Q35→S26→G2
Case Title	Wuxi City, Jiangsu Province 1.11	Baodiqu City, Tianjin Province 11.30	Hefei City, Anhui Province 07.09	Shangyu City, Zhejiang Province 1.07
Event chain	S2→Q6→Q12.	S14→G6.	Q35→G11→L4→L2	S27→S16→S21→Q10.
Case Title	Shijiahzuang City, Hebei Province 8.19	Wanzhou City, Chongqing Province 6.21	Wuhan City, Hubei Province 5.01	Haerbin City, Heilongjiang Province 7.14
Event chain	Q32→Q31→Q35	Q15→G7	S5→Q18→S14.	S26→Q36.
Case Title	Xian City, Shanxi Province 5.02	Huzhou City, Zhejiang Province 3.22	Xian City, Shanxi Province 5.02	Chaoyang City, Beijing Province 2.17
Event chain	Q10→G9	S19→G11.	S18→Q26→Q27→S25.	S26→S28→Q34→S22.
Case Title	Wuxi City, Jiangsu Province 1.11	Baodi City, Tianjin Province 11.30	Hefei City, Anhui Province 7.09	Suangyashan City, Heilongjiang Province 9.10
Event chain	G6→G7→L2	Q8→S1→L5	Q9→S2	S2→S5.
Case Title	Mianyang City, Sichuan Province 2.21	Shijiazhuang City, Hebei Province 5.31	Linfen City, Shanxi Province 4.13	Changchun City, Jilin Province 8.16
Event chain	S8→Q13→Q14.	Q37→G6	Q16→G9	Q22→G10
Case Title	Panzhihua City, Sichuan Province 7.19	Chengdu City, Sichuan Province 6.25	Jilin City, Jilin Province 9.12	Shenzhen City, Guangdong Province 9.17
Event chain	Q7→Q6→Q25	G3→G12	Q7→Q4→Q24	Q22→S13→Q35
Case Title	Zhoukou City, Henan Province 1.13	Nanjing City, Jiangsu Province 2.19		
Event chain	G1→L1→L2	Q35→S26→G2		
